# Studying the impact of titanium dioxide nanoparticles on the expression of pivotal genes related to menthol biosynthesis and certain biochemical parameters in peppermint plants (*Mentha Piperita* L.)

**DOI:** 10.1186/s12870-024-05228-9

**Published:** 2024-06-11

**Authors:** Fatemeh Ramzanpoor Veleshkolaii, Mahyar Gerami, Elham Younesi-Melerdi, Masoumeh Rezaei Moshaei, Saeed Ghanbari Hassan Kiadeh

**Affiliations:** 1 Department of Horticulture, Sana Institute of Higher Education, Sari, Iran; 2 Department of Biology, Faculty of Sana Institute of Higher Education, Sari, Iran; 3 Genetics and Agricultural Biotechnology Institute of Tabarestan, Sari Agricultural Sciences and Natural Resources University, Sari, Iran; 4 Department of Biotechnology, Amol University of Special Modern Technologies (AUSMT), Amol, Iran

**Keywords:** Catalase, Guaiacol peroxidase, Geranyl diphosphate synthase, Menthone reductase, Menthofuran synthase, RT-qPCR

## Abstract

**Background:**

This study examines the impact of titanium dioxide nanoparticles (TiO_2_NPs) on gene expression associated with menthol biosynthesis and selected biochemical parameters in peppermint plants (*Mentha piperita* L.). Menthol, the active ingredient in peppermint, is synthesized through various pathways involving key genes like geranyl diphosphate synthase, menthone reductase, and menthofuran synthase. Seedlings were treated with different concentrations of TiO_2_NPs (50, 100, 200, and 300 ppm) via foliar spray. After three weeks of treatment, leaf samples were gathered and kept at -70 °C for analysis.

**Results:**

According to our findings, there was a significant elevation (*P* ≤ 0.05) in proline content at concentrations of 200 and 300 ppm in comparison with the control. Specifically, the highest proline level was registered at 200 ppm, reaching 259.64 ± 33.33 µg/g FW. Additionally, hydrogen peroxide and malondialdehyde content exhibited a decreasing trend following nanoparticle treatments. Catalase activity was notably affected by varying TiO_2_NP concentrations, with a significant decrease observed at 200 and 300 ppm compared to the control (*P* ≤ 0.05). Conversely, at 100 ppm, catalase activity significantly increased (11.035 ± 1.12 units/mg of protein/min). Guaiacol peroxidase activity decreased across all nanoparticle concentrations. Furthermore, RT-qPCR analysis indicated increased expression of the studied genes at 300 ppm concentration.

**Conclusions:**

Hence, it can be inferred that at the transcript level, this nanoparticle exhibited efficacy in influencing the biosynthetic pathway of menthol.

**Graphical Abstract:**

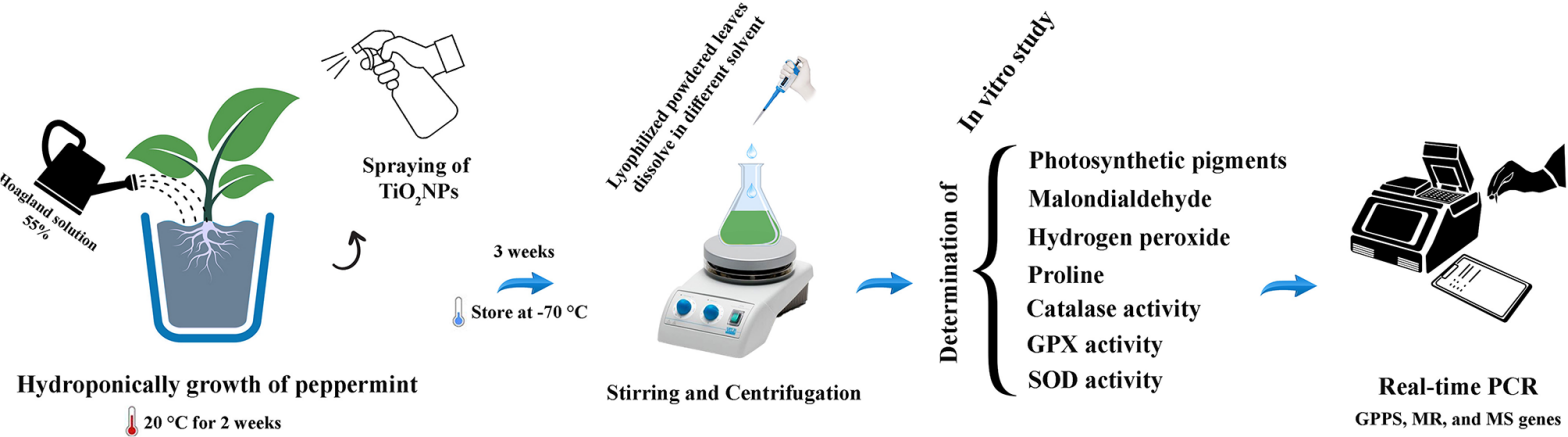

## Background

In recent times, increasing concerns regarding the adverse effects of chemical medications have shifted focus towards alternative remedies. Among researchers, a paramount objective is to discover herbal medicines that are efficacious and devoid of side effects for treating various ailments [1]. Extensive investigations into herbal plants and medications containing natural active ingredients have catalyzed a paradigm shift in medical science and pharmacy. Approximately 30% of medications consumed in human societies are derived from plants. Research has demonstrated that bioactive compounds present in these plants can ameliorate or even prevent a diverse array of diseases [2, 3]. Peppermint (*Mentha piperita* L.) is among the herbal plants garnering significant interest from researchers owing to its multifaceted therapeutic characteristics. These include antibacterial, anti-inflammatory, antifungal, anti-flatulent, and pain-relieving properties for irritable bowel syndrome (IBS) [4]. Additionally, it finds application as a sweetening agent in the food industry. These effects are primarily assigned to peppermint essential oil (EO), a component of its secondary metabolites (SMs). In many instances, the abundance of secondary metabolites (SMs) in plants relies on compounds that may not be pivotal in various life stages of the plants but hold significance in their interaction with the environment [5]. Hence, every factor impacting the quantity and quality of essential oil (EO) is thoroughly investigated [6]. Monoterpenes stand out as primary constituents of peppermint EO, with menthol and menthone being the most notable. The menthol content in peppermint serves as a key criterion for assessing EO quality [7]. Employing suitable nanoparticles presents an intriguing avenue for enhancing the stability and efficacy of these metabolites.

Nanoparticles possess distinctive physical and chemical attributes such as biocompatibility, solubility, surface charge, quantum effects, and aggregation, which are primarily contingent upon the composition, size, and shape of the particles [8, 9]. Utilization of nanoparticles comprising various elements has been explored in agriculture to promote sustainable crop production by mitigating diseases, minimizing nutrient losses, and augmenting the yield of active plant compounds [10]. These nanoparticles penetrate plants via stomatal pores and are subsequently distributed to different tissues. Moreover, plants exhibit varying responses to nanoparticles based on factors such as age, species, and inherent characteristics [11].

Titanium dioxide nanoparticles (TiO_2_NPs) have gained widespread application in both agricultural and food sectors owing to their protective functions against light and their ability to enhance plant growth, photosynthesis, and chlorophyll levels [12]. These nanoparticles bolster the efficacy of SMs by amplifying chloroplast photophosphorylation activity and photosystem II photoreduction, thereby promoting oxygen release. Additionally, they modulate the activity of nitrate reductase, rubisco, catalase, and peroxidase enzymes, while also augmenting the levels of certain essential elements in plant tissues. Research indicates that titanium promotes growth stimulation and facilitates the absorption of crucial elements like calcium, nitrogen, magnesium, manganese, phosphorus, iron, and zinc [13]. These intracellular alterations initiate diverse signaling pathways to translate physical stress into an appropriate biochemical response, each impacting the expression of stress-responsive genes [14]. The activation of these pathways facilitates plant adaptation and enhances its resilience against stressors [15]. Analyzing gene expression patterns can provide valuable insights into how plants react to nanoparticle exposure, thereby aiding in the development of effective strategies to enhance plant performance.

This study explores the impact of TiO_2_NPs on the expression of genes crucial for menthol synthesis pathway, including geranyl diphosphate synthase (GPPS), menthone reductase (MR), and menthofuran synthase (MS), alongside examining the biochemical characteristics of peppermint.

## Methods

### Material and treatments

Trichloroacetic acid, thiobarbituric acid, sulfosalicylic acid, blue tetrazolium chloride, and potassium iodide were procured from Sigma-Aldrich Company, USA. Toluene, sodium carbonate, methanol, and hydrogen peroxide were obtained from Merck Company, Germany.

### Growth conditions and treatment

Peppermint seedlings were hydroponically cultivated in a growth medium comprising perlite, cocopeat, and peat moss (in 1:2:1 v/v ratio) under a light-dark photoperiod (8 h dark/16 h light) at 55% humidity and 20 °C. Irrigation was carried out every other day using the Hoagland solution. Following a 2-week cultivation period, various concentrations of TiO_2_NPs (50, 100, 200, and 300 ppm) were applied to the seedlings as a foliar spray. After 3 weeks of treatment, leaf samples were harvested, instantly frozen using liquid nitrogen, and kept for analysis at -70 °C.

### Determination of photosynthetic pigments content

10 mg of powdered peppermint leaves were dissolved in methanol (1.5 mL) and stand at ambient temperature for 10 min. Subsequently, the product was centrifuged for 20 min at 15,000 rpm. The resulting supernatant was scrutinized using a spectrophotometer at 665.2, 652.4, and 470 nm. The quantities of total chlorophyll (total Chl), chlorophyll *a* (Chl *a*), chlorophyll *b* (Chl *b*), and carotenoids were then determined by these formulas [16].

Chl *a* (µg/mL) = 16.72 *A*_665.2_ − 9.16 *A*_652.4_ (1).

Chl *b* (µg/mL) = 34.09 *A*_652.4_ − 15.28 *A*_665.2_ (2).

Total Chl (µg/mL) = Chl *a* + Chl *b* (3).

Carotenoids (µg/mL) = (1000 *A*_470_ − 1.63 Chl *a* − 104.96 Chl *b*) /221 (4).

### Determination of malondialdehyde concentration

To quantify malondialdehyde (MDA), 0.1 g of lyophilized powdered peppermint leaves were combined with 0.1% trichloroacetic acid (TCA) (1.5 mL), followed by centrifugation for 15 min at 10,000 rpm. The resulting supernatant was preserved. Subsequently, 1 mL of 10% TCA and 5% thiobarbituric acid (TBA) were mixed with the supernatant and incubated in a water bath at 95 °C for 15 min, followed by immediate centrifugation for 5 min at 10,000 rpm. The optical density (OD) of the supernatant was then evaluated at 600 nm and 532 nm using a spectrophotometer. In this assay, 0.1% TCA served as the control sample [2].

### Determination of hydrogen peroxide concentration

For hydrogen peroxide (H_2_O_2_) measurement, lyophilized powdered leaves (0.1 g) were blended with 0.1% TCA (1.5 mL), followed by centrifugation for 15 min at 10,000 rpm. Subsequently, 1 M potassium iodide (1000 µL) and phosphate buffer (500 µL) were mixed with the supernatant, and the optical density OD was measured at 390 nm [17].

### Determination of proline content

To determine the proline content, lyophilized powdered leaves (0.1 g) were combined with cold phosphate buffer (1.5 mL) (containing 0.5 mM EDTA, pH 7.5). Following centrifuging at 15,000 rpm for 30 min, the supernatant was utilized as an enzyme extract. Its volume was adjusted to 10 mL with 3% sulfosalicylic acid and centrifuged at 10,000 g for 10 min. Subsequently, the supernatant (2 mL) was combined with pure 2 mL of acetic acid and 2 mL of ninhydrin reagent, followed by one-hour boiling. To halt the reaction, the sample was rapidly transferred to a container containing water and ice for 20 min. Afterward, toluene (4 mL) was combined with each sample and mixed. Lastly, the solution OD was measured at 520 nm. The proline concentration of the sample was specified by the proline standard curve, calculated as µg/g leaf FW [18].

### Catalase activity assay

To accomplish this, 30 µL of enzyme extract (the solution obtained in Sect. 2.6) was combined with phosphate buffer (2970 µL) containing 2 mM hydrogen peroxide (H_2_O_2_), and the OD was evaluated at 240 nm. The Beer-Lambert law was employed for determining enzyme activity with an extinction coefficient of 40 µM^− 1^cm^− 1^ [19].

### Guaiacol peroxidase (GPX) activity assay

To conduct this experiment, enzyme extract (50 µL) was blended with phosphate buffer (700 µL) and 700 µL of H_2_O_2_. Changes in optical density were monitored at 470 nm over a 2-minute period. Cold phosphate buffer served as the control sample [19].

### Superoxide dismutase activity assay

To quantify the level of superoxide dismutase (SOD), enzyme extract (100 µL) was combined with solution A (2000 µL) (consisting of 50 mM phosphate buffer, 12 mM methionine, 50 mM sodium carbonate, and 75 µM nitro blue tetrazolium chloride) and solution B (100 µL) (1 µM riboflavin). This mix was then put into a glass sample tube and exposed to a fluorescent lamp (15 W) for 10 min at a distance of 35 cm. Following the lamp’s deactivation, the reaction was terminated, and its OD was evaluated by a spectrophotometer at 560 nm. Additionally, a sample tube with the reaction mix (solutions A and B) served as the blank. One unit of SOD activity was characterized as the amount of enzyme causing a 50% decrease in the nitro blue tetrazolium photoreduction [20].

### Assessment of gene expression engaged in the biosynthesis pathway of menthol

#### RNA extraction and cDNA synthesis

Upon collection, peppermint plant leaves were promptly flash-frozen with liquid nitrogen and then pulverized using a mortar. Total RNA extraction was conducted by the use of Iraizol kits following the protocols provided by the manufacturer. The purity and concentration of the isolated RNA were assessed by the use of a nano spectrophotometer (Epoc, Biotech, USA) and 1.2% agarose gel electrophoresis, respectively. The extracted RNA underwent DNase I treatment (RNA Biotech, Co) to eliminate genomic DNA contamination, and cDNA synthesis was done utilizing the Thermo Scientific RevertAid Kit (USA) as per the manufacturer’s instructions. The resulting cDNAs were kept at -20 °C until further application [21].

### Primer design and real-time PCR conditions

Gene-specific primers (for GPPS, MR, and MS genes) were designed using Primer 3 software based on the cDNA sequences obtained from the National Center for Biotechnology Information (NCBI). Table 1 presents the amplicon characteristics and primer sequences for each gene. Real-time PCR amplification was conducted in 96-well plates using the Green Supermix Kit Eva Green SYBR (BioRad) following the manufacturer’s protocols and Quantitation-Comparative Ct (ΔΔCt) approach. Briefly, the experimental conditions comprised denaturation for 2 min at 95 °C, followed by 40 cycles of 95 °C for 20 s, 60 °C for 30 s, and 72 °C for 25 s. The beta-actin housekeeping gene served as the internal reference for normalizing expression levels [21]. Melting curve analysis was employed to verify absence of primer dimer formation and the specificity of the amplified products.

**Table 1:** The sequence of *β*-actin, MR, MS, and GPPS primers

Gene	Primer	Sequence (3’-5’)	Primer size, bp	Product size, bp
*β*-actin	F	CTACGAAGGCTACGCACTCC	20	165
	R	GCAATGTAGGCCAGCTTCTC	20	
MR	F	CGCTGTTGCTGTTGCTCACTT	21	177
	R	GTTTTGGGATGGAATGGATGTG	22	
MS	F	GAGATGTTCATGGCGCTGAC	20	187
	R	CCACTTCTGCATCGACGCC	19	
GPPS	F	ATCTCAGCCGTTCTCCTTCA	20	150
	R	GCCTTATTGGGATGGATTTCT	21	

### Statistical analysis

In this investigation, all surveys were executed using a completely randomized design. For biochemical assays, each sample was replicated three times (*n* = 3). Statistical analysis was done utilizing one-way ANOVA and Duncan’s test. Data analysis and computations were conducted using SPSS 26 and GraphPad Prism 9 software, with a significance level as 5% (*P* ≤ 0.05).

## Results

### Photosynthetic pigment content

**Fig. 1:**
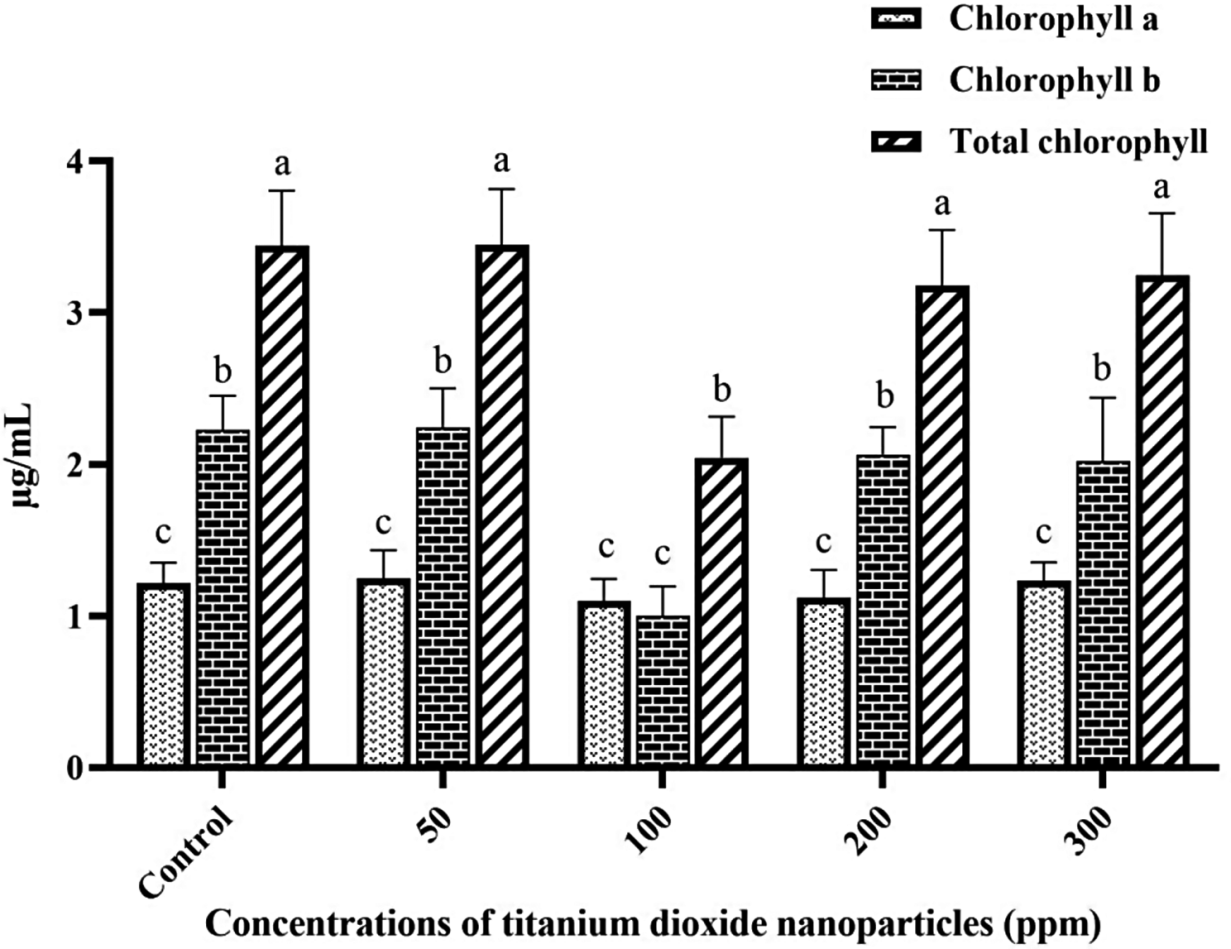
The effect of different concentrations of TiO_2_NPs on Chl *a*, Chl *b*, and total Chl of the peppermint plant. (Columns with different letters indicate significant differences at P-value < 0.05)

**Fig. 2:**
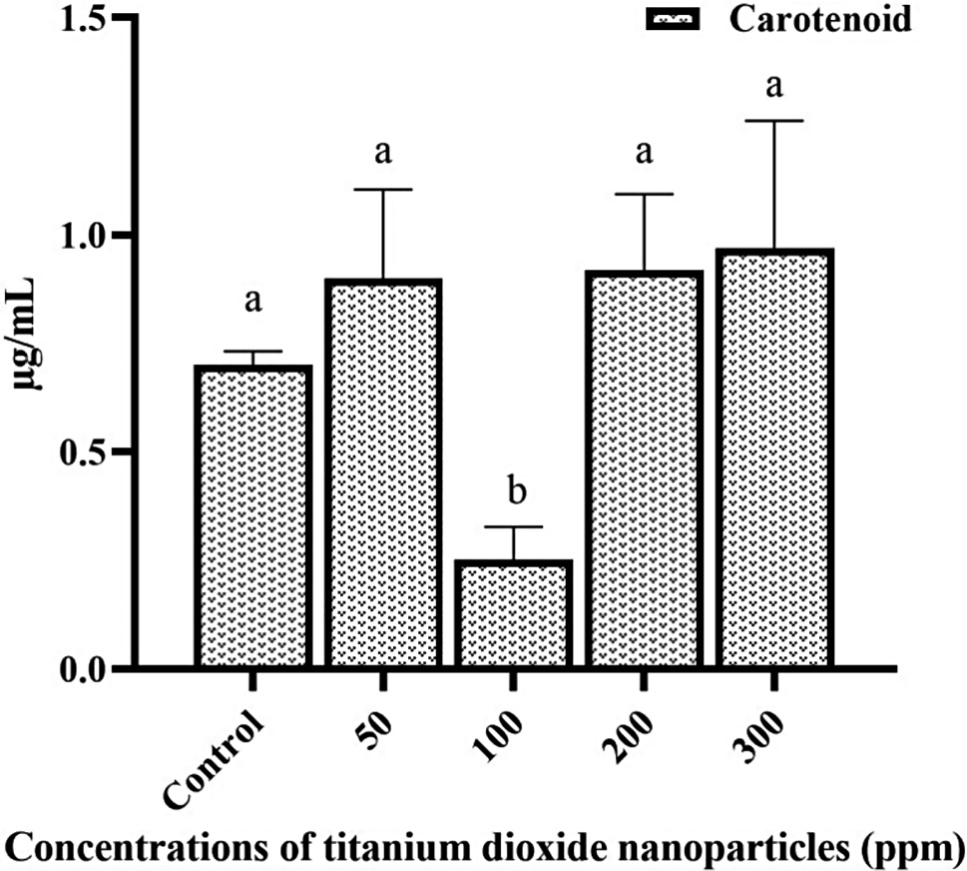
The effect of different concentrations of TiO_2_NPs on carotenoid content in peppermint plant. (Columns with different letters indicate significant differences at P-value < 0.05)

As depicted in Fig. 1, the investigation revealed that the concentration of Chl *a* in peppermint plants subjected to various TiO_2_NP concentrations did not exhibit significant alterations (*P* ≤ 0.05) in comparison with the control group. Furthermore, the findings indicated a noteworthy (*P* ≤ 0.05) decline in Chl *b* content specifically at the 100 ppm concentration, compared to both the control and other treatments (0.95 ± 0.13 µg/mL). The total Chl content displayed a significant (*P* ≤ 0.05) discrepancy solely at the 100 ppm concentration in comparison to the control (2.04 ± 0.15 µg/mL). Moreover, variations in carotenoid content indicated a significant (*P* ≤ 0.05) decline at the 100 ppm concentration in comparison to the control group, while other treatment groups did not exhibit significant differences. Figure 2 illustrates the alterations in carotenoid content in peppermint treated with various concentrations of TiO_2_NPs.

### MDA, H_2_O_2,_ and proline content

**Fig. 3:**
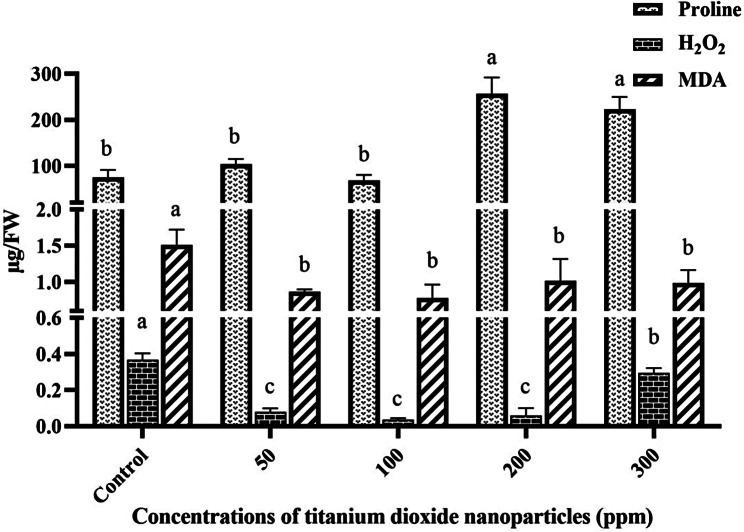
The effect of different concentrations of TiO_2_NPs on MDA, H_2_O_2,_ and proline content in peppermint plant. (In each group, columns with different letters indicate significant differences at P-value < 0.05)

According to the findings, the MDA content decreased across all examined treatments compared to the control. However, there was not any significant (*P* ≤ 0.05) difference in various concentrations. Moreover, it was noted that the H_2_O_2_ content significantly (*P* ≤ 0.05) diminished in the existence of different TiO_2_NP concentrations in comparison with the control group. Particularly, at a concentration of 300 ppm, the H_2_O_2_ content displayed a notable (*P* ≤ 0.05) alteration compared to other concentrations. Additionally, the results indicated a significant (*P* ≤ 0.05) elevation in proline content at concentrations of 200 and 300 ppm relative to the control group. Specifically, the highest proline amount at the 200 ppm concentration was recorded at 259.64 ± 33.33 µg/g FW. Figure 3 illustrates the impact of varying TiO_2_NP concentrations on MDA, H_2_O_2_, and proline content in peppermint.

### Catalase activity

**Fig. 4:**
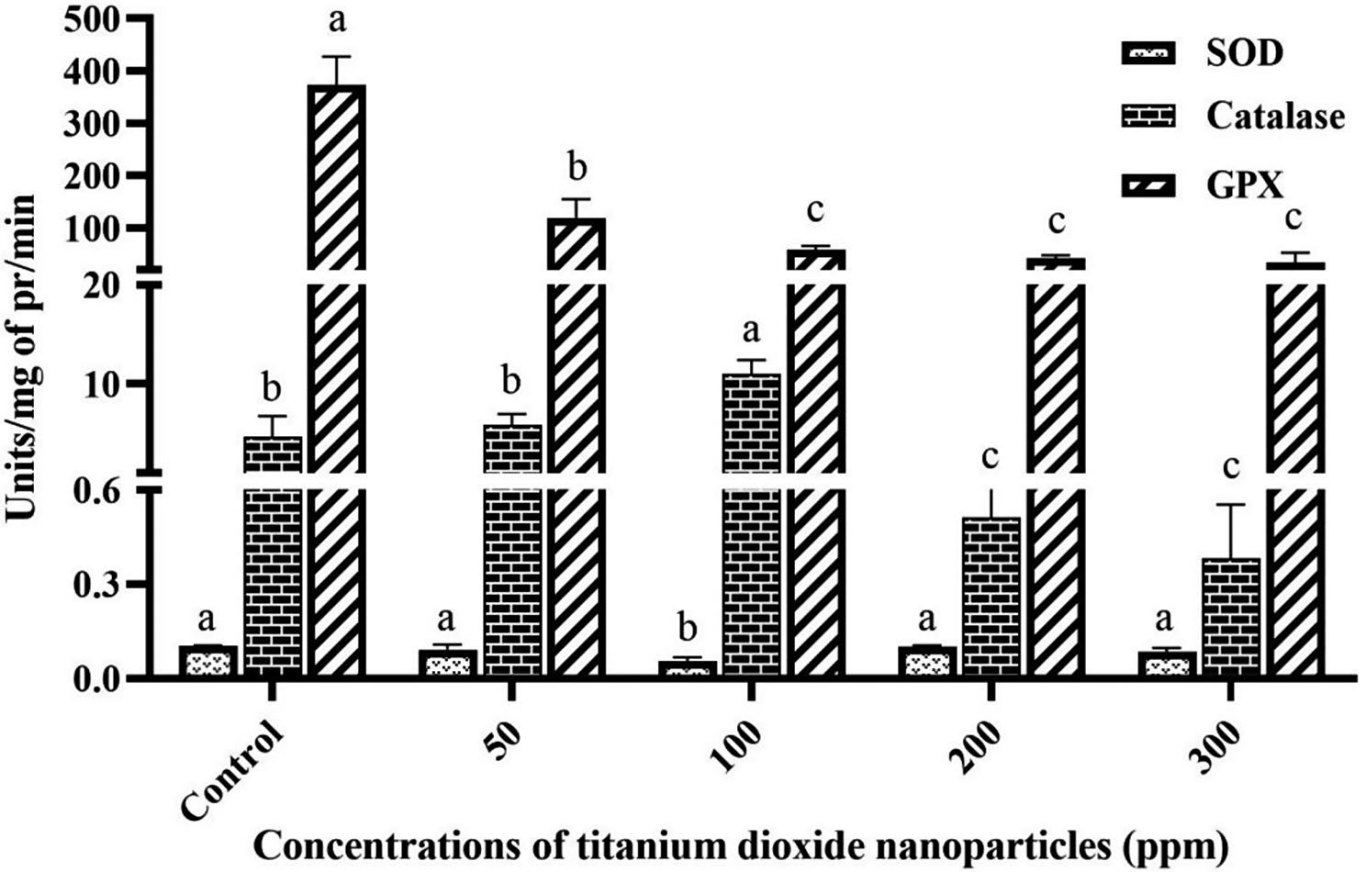
The effect of different concentrations of TiO_2_NPs on SOD, GPX, and catalase activity in peppermint plant. (In each group, columns with different letters indicate significant differences at P-value < 0.05)

The findings of this study revealed that catalase activity was impacted by varying concentrations of TiO_2_NP. Specifically, enzyme activity notably decreased (*P* ≤ 0.05) at 200 and 300 ppm concentrations in comparison to the control group. Conversely, at the 100 ppm concentration, catalase activity exhibited a significant increase (11.035 ± 1.12 units/mg of protein/min). Furthermore, there was not any significant (*P* ≤ 0.05) alteration at the 50 ppm concentration in comparison with control group. Figure 4 illustrates the influence of different TiO_2_NP concentrations on catalase activity.

### GPX activity

According to the results, the GPX activity showed a significant reduction (*P* ≤ 0.05) in all treatments in comparison with the control. The lowest enzyme activity was recorded at the concentration of 200 ppm, measured at 41.7 ± 5.2 units/mg of protein/min. Figure 4 illustrates the impact of various TiO_2_NP concentrations on GPX activity.

### SOD activity

As depicted in Fig. 4, it was observed that the level of SOD enzyme activity significantly (*P* ≤ 0.05) reduced solely in the 100 ppm treatment compared to the control group (0.54 ± 0.01 units/mg of protein/min). Conversely, other treatments did not exhibit significant (*P* ≤ 0.05) alterations in SOD activity compared to the control. The influence of various TiO_2_NP concentrations on SOD activity is illustrated in Fig. 4.

### Expression analysis of GPPS, MR, and MS genes

**Fig. 5:**
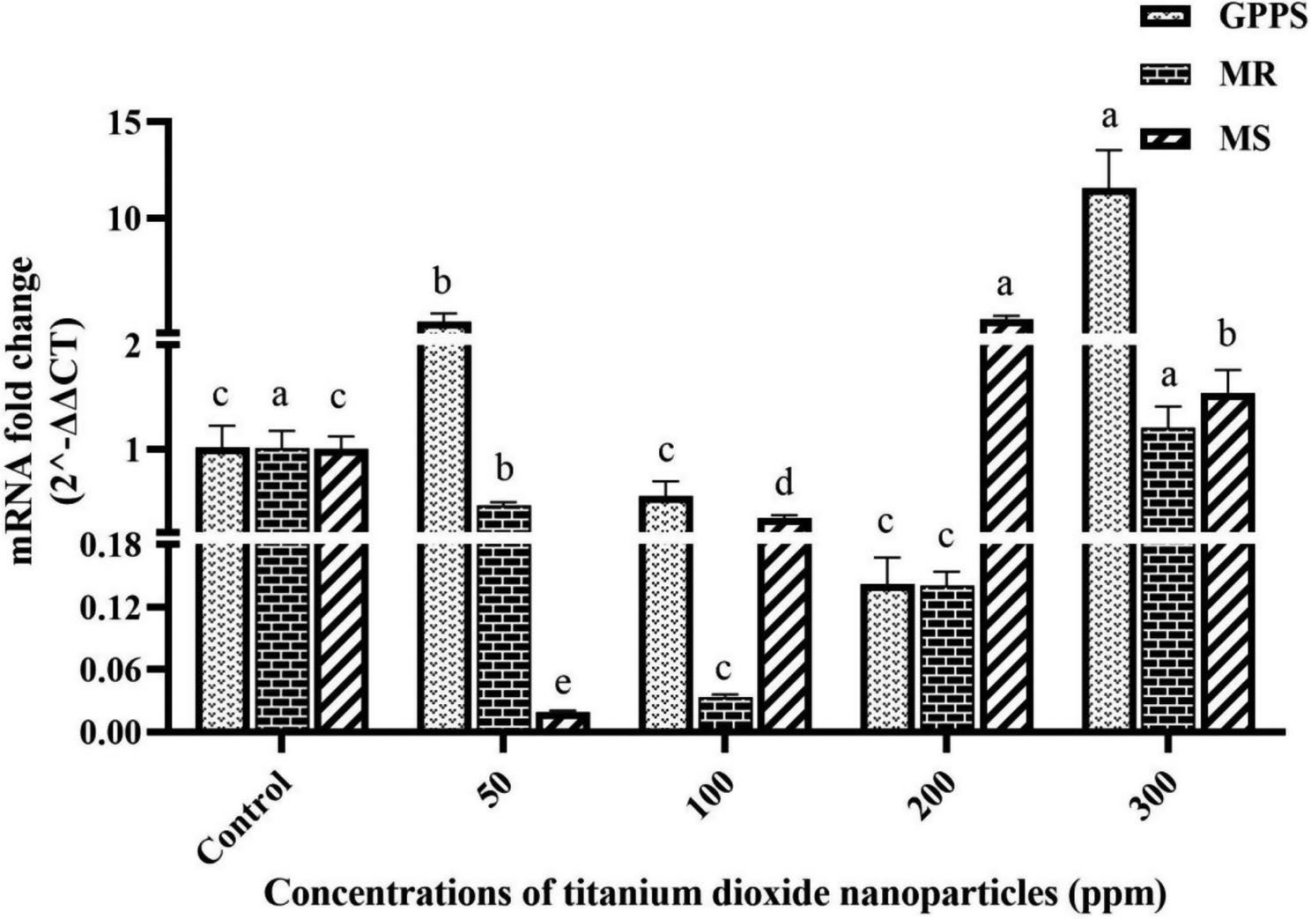
The relative expression level of GPPS, MR, and MS genes in peppermint plants under different TiO_2_NPs treatments. (In each gene, columns with different letters indicate significant differences at P-value < 0.05)

The RT-qPCR analysis showed that the GPPS gene expression significantly (*P* ≤ 0.05) increased in peppermint treated with TiO_2_NP at 50 and 300 ppm concentrations in comparison with the control, showing fold changes of 4.6 and 11.56 respectively. Nevertheless, there was not any significant (*P* ≤ 0.05) alteration in expression at concentrations of 100 and 200 ppm compared to the control. Moreover, the MR gene expression was significantly (*P* ≤ 0.05) reduced at concentrations of 50, 100, and 200 ppm compared to the control, with the lowest level observed at the 100 ppm concentration (0.034-fold change compared to the control). Additionally, the MS gene expression significantly (*P* ≤ 0.05) varied across different treatments in comparison with the control. Expression levels increased at concentrations of 200 and 300 ppm (4.73 and 1.54 fold respectively), while they decreased at concentrations of 50 and 100 ppm (0.02 and 0.33 fold respectively). Figure 5 illustrates the relative expression levels of GPPS, MR, and MS genes in peppermint plants under different TiO_2_NPs treatments.

## Discussion

Recently, there has been increasing interest among researchers in harnessing nanoparticles as non-biological elicitors to manipulate SMs in different herbal plants, like peppermint. Nanoparticles, owing to their unique physicochemical properties, have the capability of augmenting the activity of bioactive compounds in plants [10]. In the current work, we assessed the impact of varying concentrations of TiO_2_NPs on peppermint plants at biochemical, molecular, and physiological levels. One of the parameters investigated at the physiological level was the alteration in photosynthetic pigments. Chlorophyll (Chl), being a crucial component in photosynthesis, converts solar energy into chemical energy [22]. The findings of this study revealed that the total Chl content remained relatively stable across varying concentrations of TiO_2_NPs, with no significant (*P* ≤ 0.05) alterations observed. This outcome in in consistency with the findings of Larue et al. (2012) [23], who investigated the impact of TiO_2_NPs on Chl levels in wheat plants. Conversely, Ahmad et al. (2018) [10] presented an elevation in total Chl content in peppermint in response to TiO_2_NPs treatment. Additionally, there are reports indicating a decline in Chl content under TiO_2_NPs treatment. Mohammadi et al. (2016) observed a decline in Chl content at a concentration of 10 ppm TiO_2_NPs in dragonhead (*Dracocephalum Moldavia* L.) plants [24]. Such discrepancies may be attributed to various factors including genetic and environmental variations among species, the specific plant parts studied, growth conditions, treatment duration, sampling methods, and nanoparticle synthesis processes [25, 26].

Metal ions such as titanium have been implicated in triggering oxidative stress within plant cells. To combat this stress, plants deploy both non-enzymatic antioxidant systems, which include flavonoids, carotenoids, and proline, as well as enzymatic antioxidant systems like catalase, SOD, and GPX [27]. Previous research has indicated that TiO_2_NPs may enhance water and phosphorus absorption while also boosting the activity of non-enzymatic and enzymatic antioxidants [28]. Carotenoids, a class of pigments, exhibit noteworthy antioxidant properties. Epidemiological investigations have demonstrated that consuming vegetables rich in carotenoids can mitigate the risk of cardiovascular diseases and certain cancers [29]. The findings of our study revealed an increase in carotenoid content across various concentrations (except for 100 ppm concentration) compared to the control, although this increase was not significant statistically (*P* ≤ 0.05). This finding closely aligns with the outcomes presented in the work performed by Gohari et al. (2020) that explored the impact of TiO_2_NPs on the biochemical attributes of *Dracocephalum Moldavia* L [30]. It has been established that carotenoids not only absorb light but also directly neutralize free radicals [31].

Proline, an amino acid found in the cytoplasm, serves a crucial role in safeguarding intracellular macromolecules against environmental stresses, including heavy metal exposure. The results indicated a significant (*P* ≤ 0.05) rise in proline levels under the treatment of 200 and 300 ppm of TiO_2_NPs. This increase in proline content signifies the activation of osmotic regulation mechanisms, facilitating enhanced absorption of water and solutes from the surroundings [32]. A study by Mazarie et al. (2019) [33] observed a similar trend, where the dissolution of TiO_2_NPs led to increased proline content in sage (*Salvia officinalis* L.) under conditions of water stress.

As previously discussed, plants counteract reactive oxygen species (ROS) by enzymatically converting them into hydrogen peroxide (H_2_O_2_) via superoxide dismutase (SOD) synthesis. ROS production often inflicts damage on cell membranes [34]. This damage can be assessed by measuring malondialdehyde (MDA) production. The findings of this study demonstrated that various concentrations of TiO_2_NPs effectively scavenged H_2_O_2_ from the cellular environment, resulting in a significant (*P* ≤ 0.05) reduction in MDA content. A study by [35] observed a similar trend in Rosmarinus officinalis L., where increasing TiO_2_NP concentration led to decreased H_2_O_2_ levels. This reduction is likely attributable to enhanced antioxidant enzyme activity, prompting an investigation into the activity levels of certain enzymes in this study. The results indicated that SOD activity did not significantly (*P* ≤ 0.05) differ from the control. However, catalase enzyme activity increased at a concentration of 100 ppm, while it decreased at concentrations of 200 and 300 ppm. Hence, it can be inferred that TiO_2_NPs function as an elicitor, enhancing catalase activity up to a concentration of 100 ppm, with this effect diminishing at higher concentrations. This observation aligns with findings by Khalilvand et al. (2019), who noted a reduction in catalase activity with increasing TiO_2_NP concentration in sweet corn [36]. Additionally, the activity of the GPX enzyme exhibited a significant (*P* ≤ 0.05) decrease across all concentrations. Consequently, it is speculated that the augmentation in antioxidant activity induced by various TiO_2_NP treatments predominantly relies on the non-enzymatic antioxidant system, particularly the notable (*P* ≤ 0.05) rise in proline levels. Moreover, research has indicated that H_2_O_2_ can also be scavenged by other enzymes such as ascorbate-peroxidase (APX) [30].

Menthol, a key monoterpene in peppermint essential oil (EO), significantly enhances EO efficiency [37]. Elicitors have been shown to boost the biosynthesis of SMs by activating specific genes [38]. Thus, following treatment with varied TiO_2_NP concentrations, the expression of GPPS, MR, and MS genes crucial for menthol biosynthesis was examined in peppermint. Results revealed that the 300 ppm TiO_2_NP concentration elevated GPPS expression. Additionally, MS expression increased at 200 and 300 ppm, whereas it decreased at lower concentrations. Thus, higher TiO_2_NP concentrations may upregulate these genes and potentially boost menthol production as an elicitor. Furthermore, MR expression significantly (*P* ≤ 0.05) decreased across all concentrations except 300 ppm. While no prior study has specifically investigated TiO_2_NP effects on this pathway, our findings suggest that these nanoparticles alter gene expression involved in menthol synthesis.

## Conclusion

The distinctive physicochemical properties of nanoparticles elicit diverse responses in plants. TiO_2_NPs, for instance, can enhance enzyme activities, augment bioactive compound levels, and improve the functional traits of *Mentha piperita* L. The findings of this study revealed that varied TiO_2_NP concentrations significantly (*P* ≤ 0.05) impacted the antioxidant system of *Mentha piperita* L. They caused a significant (*P* ≤ 0.05) decline in MDA and H_2_O_2_ levels and considerable increase in proline content, while total Chl content remained relatively unchanged. Catalase activity saw an increase at 100 ppm, whereas GPX activity decreased across all concentrations. Moreover, the TiO_2_NP effect on the expression of genes engaged in menthol synthesis supports their role as elicitors in peppermint plants. These nanoparticles hold promise as inducers for secondary metabolite production. Nevertheless, further investigation is warranted to elucidate the molecular mechanisms underlying these effects.

## Data Availability

The data supporting the study’s findings are accessible from the authors upon reasonable request.
